# Creating leadership collectives for sustainability transformations

**DOI:** 10.1007/s11625-021-00909-y

**Published:** 2021-03-04

**Authors:** O. Care, M. J. Bernstein, M. Chapman, I. Diaz Reviriego, G. Dressler, M. R. Felipe-Lucia, C. Friis, S. Graham, H. Hänke, L. J. Haider, M. Hernández-Morcillo, H. Hoffmann, M. Kernecker, P. Nicol, C. Piñeiro, H. Pitt, C. Schill, V. Seufert, K. Shu, V. Valencia, J. G. Zaehringer

**Affiliations:** 1 The Careoperative, Berlin, Germany; 2 School for the Future of Innovation in Society, Arizona State University, Tempe, AZ 85281 USA; 3 Department of Geography and URPP Global Change and Biodiversity, University of Zurich, Winterthurerstrasse 190, 8057 Zurich, Switzerland; 4 Faculty of Sustainability, Leuphana University of Lüneburg, Universitätsallee 1, 21335 Lüneburg, Germany; 5 Department of Ecological Modelling, Helmholtz Centre for Environmental Research–UFZ, Permoserstr. 15, 04318 Leipzig, Germany; 6 Institute of Environmental Systems Research, University of Osnabrück, Barbarastr. 12, 49076 Osnabrück, Germany; 7 Department of Ecosystem Services, Helmholtz Centre for Environmental Research–UFZ, Puschstrasse 4, 04103 Leipzig, Germany; 8 Department of Ecosystem Services, German Center for Integrative Biodiversity Research (iDiv) Halle-Jena-Leipzig, Puschstrasse 4, 04103 Leipzig, Germany; 9 IRI THESys, Humboldt-Universität Zu Berlin, Unter den Linden 6, 10099 Berlin, Germany; 10 Section for Geography, Department of Geosciences and Natural Resource Management, University of Copenhagen, Øster Voldgade 10, 1350 Copenhagen K, Denmark; 11 School of Geography and Sustainable Communities, University of Wollongong, Wollongong, 2522 Australia; 12 Department of Agricultural Economics and Rural Development, University of Goettingen, Platz der Göttinger Sieben 5, 37073 Göttingen, Germany; 13 Stockholm Resilience Centre, Stockholm University, 106 91 Stockholm, Sweden; 14 Faculty of Forest and Environment, Eberswalde University for Sustainable Development, Alfred Möller Straße 1, 16225 Eberswalde, Germany; 15 Leibniz Centre for Agricultural Landscape Research (ZALF), Eberswalder Straße 84, 15374 Müncheberg, Germany; 16 Sustainable Places Research Institute Cardiff University, 33 Park Place Cardiff, Wales, CF10 3BA UK; 17 Altekio S.Coop.Mad, Paseo de Las Acacias, 3, 1a, 28005 Madrid, Spain; 18 Beijer Institute of Ecological Economics, Royal Swedish Academy of Sciences, Stockholm, Sweden; 19 Institute for Environmental Studies (IVM), Vrije Universiteit Amsterdam, De Boelelaan 1111, 1081 HV Amsterdam, The Netherlands; 20 Institute of Soil Science and Plant Cultivation State Research Institute, Czartoryskich 8 Street, 24-100 Puławy, Poland; 21 Farming Systems Ecology Group, Wageningen University and Research, 6700AK Wageningen, The Netherlands; 22 Centre for Development and Environment, University of Bern, Mittelstrasse 43, 3012 Bern, Switzerland

**Keywords:** Sustainability transition, Collegiality, Well-being, Equality, Academic practice

## Abstract

Enduring sustainability challenges requires a new model of collective leadership that embraces critical reflection, inclusivity and care. Leadership collectives can support a move in academia from metrics to merits, from a focus on career to care, and enact a shift from disciplinary to inter- and trans-disciplinary research. Academic organisations need to reorient their training programs, work ethics and reward systems to encourage collective excellence and to allow space for future leaders to develop and enact a radically re-imagined vision of how to lead as a collective with care for people and the planet.

**Supplementary Information:**

The online version contains supplementary material available at 10.1007/s11625-021-00909-y.

## Introduction

The latest scholarship and training programs in sustainability science and practice recognise the importance of collective leadership for addressing pressing sustainability challenges. Yet, the focus of these efforts is on centralised models of collective leadership in which an individual leader is responsible for crossing boundaries and establishing collaborative partnerships to transform systems (e.g. Gordon et al. 2019). Such models are evident in the Earth Leadership Program (www.earthleadership.org/) and the Homeward Bound program (https://homewardboundprojects.com.au/), where the foci are on building the capabilities of individuals to convene groups and develop shared visions. In our view, such collective leadership models remain insufficient to enact meaningful and equitable sustainability transformations. A polycentric form of collective leadership is needed to achieve structural changes. We advocate for the creation of leadership collectives: groups of individuals from multiple organisations and sectors who lead transformational social change together through critical reflection, inclusivity and care.

Leadership collectives require *critical reflection* to transform how collective leadership is defined and embodied; to challenge the existing structures in sustainability science and practice that re-inforce problematic leadership ideals; and to respond to the complexity and uncertainties of sustainability transformations.

*Inclusivity* is required to remedy the systematic marginalization of people (whether because of gender, ability, racial or class constructs, etc.) excluded by traditional leadership models. Inclusivity contributes to critical reflection by broadening the diversity of perspectives, ideas and styles of leadership, allowing for thoughtful exchange.

*Care* has the potential to be the most transformative aspect of leadership collectives. Well described by a collegiate caring collective at the University of Newcastle in Australia, attending to care “involves the mutual recognition of an individual’s situation, active listening, the development of trust, and ongoing expressions of solidarity” (Ey et al. 2020). Similarly, a focus on care for the planet and people (Corbera et al. 2020) is essential both for sustaining leadership collectives and sustainability transformations requiring long-term engagements and partnerships beyond an individual's career or an organisation’s existence.

Over the past 2 years, we have been part of a professional development program^1^ training early-career scholars in leadership competencies for sustainability. Through this program, we have connected with international researchers in our academic cohort and since synthesised the opportunities, gaps and critical needs for our field going forward. Our experiences as a cohort have foregrounded the need for an alternative model of leadership, based on critical reflection, inclusivity and care, which focuses attention beyond forging individual leaders for sustainability science organisations (as per Gordon et al. 2019; Boone et al. 2020). We expand below on a broader, more holistic view of fostering *leadership collectives* to facilitate sustainability within academia and society.

## Reorienting academia to drive structural change

The current academic system discourages the type of leadership required for sustainability transformations. Systemic foci on output-based metrics and internationally mobile careers favour individuals able to pursue prestige and promote personal excellence within specific disciplines (Coate and Howson 2016). A recent global survey found that 78% of researchers think competition in academia has created unkind, aggressive working conditions and 75% said creativity is stifled (Shift Learning 2020). Structural changes are needed if academia is to provide conditions that encourage leadership collectives to emerge and embrace critical reflection, inclusivity and care as a way to enable sustainability transformations. We identify three systemic patterns that need to change:

*From metrics to merits* Research excellence is currently almost exclusively evaluated on individual output-based metrics (e.g. number of first or last-author publications or grant income secured as principal investigator) (Wilsdon et al. 2015). This triggers and re-inforces unhealthy competition and disadvantages individuals who invest in long-term collaborative processes crucial to transformative research.

To foster leadership collectives, the measurement of scientific excellence needs to acknowledge and reward collaborative merits that often require more time and resources. Such merits ought to account for researchers’ investment in enabling inclusive and trustful collective action, e.g. coordinating transdisciplinary processes for societally-relevant research, knowledge brokering, and building long-term and diverse research partnerships with communities.

*From career to care* Progressing as a leader in sustainability science, as in any other research field, often requires individuals to sacrifice work–life balance at the expense of wellbeing (Shift Learning 2020). This requirement, together with expectations that leaders are flexible and internationally mobile, penalises individuals who have and want to prioritise caring responsibilities for family, friends, community, and place (Pugh and Thomas 2021; Manzi et al. 2019). Perpetual career impermanence, often experienced most acutely in early-career phases, undermines the ability to form long-term collaborations. De-prioritisation of care squeezes scientifically-talented women and minorities out of the ‘leaky pipeline’, leaving a homogeneous cadre of leaders, and limits advancement of those favouring cooperative leadership styles (Grummell et al. 2009; Coate and Howson 2016). Career roadblocks and the personal sacrifices required for overcoming these are often higher for people of colour and other minorities (Johnson and Joseph-Salisbury 2018; Montgomery 2020).

Supporting working carers (e.g. through family-friendly work practices and facilities) and rewarding caring as a valuable leadership attribute will make senior roles more accessible, inclusive and healthy (Grummell et al. 2009). More diverse leadership options, such as job-share professorships, could also provide more space for caring in leadership collectives.

*From inter- and trans-disciplinarity on paper to practice* Despite long-standing calls for and investment in more interdisciplinary research across sustainability sciences and transdisciplinary research with society, funding remains heavily structured around disciplines and sectors. Sustainability research is still largely delivered by people with strong disciplinary roots, and discussions in different fields often happen in parallel without cross-pollination (Haider et al. 2018). Although trans-disciplinarity is often seen as an important prerequisite for transformative sustainability research, true trans-disciplinarity faces multiple barriers within current academic systems (Jordan et al. 2016), such as a lack of common research framing (Brandt et al. 2013), unbalanced problem ownership (Lang et al. 2012), and methodological conflicts (Pohl and Hadorn 2008).

Leadership collectives need to cross boundaries between disciplines, and between academia and society. Bridging these boundaries requires leaders with specific skills, including epistemological agility, knowledge brokering, creativity and self-reflexivity (Haider et al. 2018). There is a need for educational and training programs that develop such capabilities among disciplinarily diverse cohorts and for further investment in funding programs that recognise the unique challenges facing inter- and trans-disciplinary research.

Together, these suggestions imply reorienting academia away from maximising individual outputs in minimal time, towards a slower (more sustainable) science with time to centre wellbeing and change processes, and hold space to be creative and collaborative (Mountz et al. 2015; Stengers 2018).

## The Careoperative: a leadership collective experiment

These aspirations for a reorientation of academia are reflected in our motivations for experimenting with a new leadership collective, the Careoperative, which brings together a group of individuals from multiple organisations and disciplines in collaborations beyond research projects. The name Careoperative conveys our common goal of providing a reflexive, inclusive and caring space for members as we pursue our mission to collectively explore, embody and lead transformational sustainability research and practice.

**Fig. 1:**
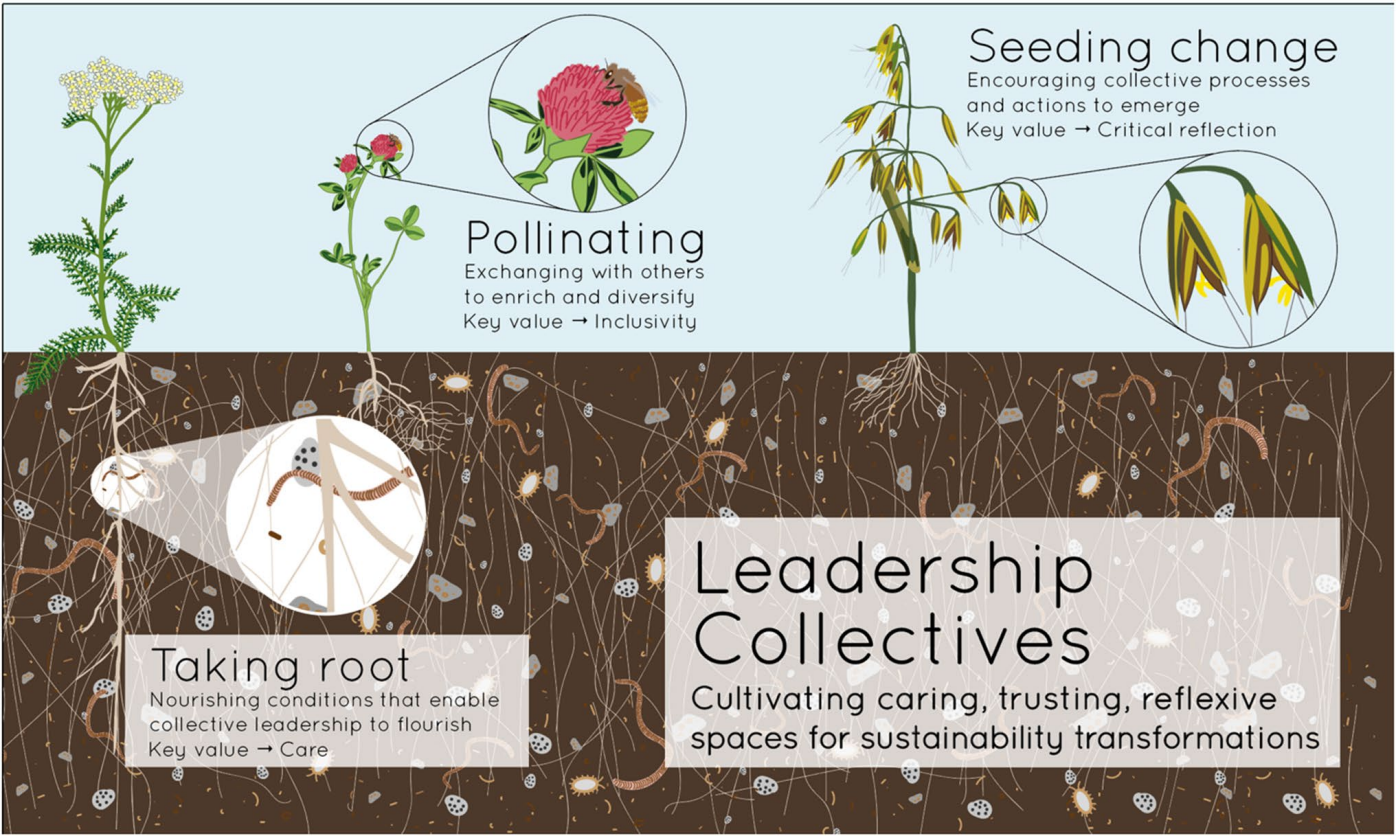
A metaphorical representation of leadership collectives. Analogous to healthy agro-ecosystems, leadership collectives require nourishing conditions (taking root), exchanging with others to enrich and diversify (Pollinating), and encouraging collective processes and action to expand (Seeding change), to cultivate caring, trusting and reflexive spaces for sustainability transformations. (Figure by: Veronica Remmele)

As a living experiment starting in October 2019, the Careoperative provides a space of support for sharing professional and personal experiences, connecting different perspectives and positions on sustainability transformation, and developing collective leadership skills through self-organisation, distributed responsibility and mutual respect. While growing out of relationships established through in-person meetings, we have used regular virtual meetings and shared online workspaces to expand and deepen our collaboration. When the COVID-19 pandemic hit in the beginning of 2020, these regular virtual interactions provided a strong collegial support system that enabled us to maintain both our collaborative work and provide peer support to deal with the new challenges of, for instance, balancing work–life and care duties or conducting research in foreign contexts (The Care Operative 2020). In various ways, the Careoperative is distinguishable from other professional networks we take part in by its aim to achieve “more-than-outputs”. The Careoperative instead presents a seed of change from which we draw inspiration and support to explore transforming our research practices and work environments in the present and with eyes to our future careers within and beyond academia. We see three key ways through which the Careoperative is emerging as an invaluable foundation upon which to develop collective and transformative research leadership (see Fig. 1).

*Taking root—nourishing conditions that enable collective leadership to flourish* The Careoperative provides nourishing conditions—an ethic of care, based upon egalitarian ways of working and shared responsibility—that support us to develop roots and flourish. An ethic of care is often invoked with regard to earth stewardship (West et al. 2018). In the Careoperative, caring for ourselves and each other as colleagues working towards sustainability transformations forms part of our broader endeavour of caring for the planet and society (Corbera et al. 2020). We use non-hierarchical and non-competitive ways of working. Our choice of writing under a shared first-author pseudonym, O. Care, for example, reflects how this paper is the result of a collective effort, whilst challenging lead author status as indication of prestige. A first-author pseudonym with alphabetical contributor listing recognizes the varied but essential contributions of the entire collective to the processes of learning, reflection and writing that have resulted in this paper. Sharing responsibility provides opportunities to develop and practice transformational leadership skills, including facilitation and coordination, while accommodating diverse caring responsibilities. Sharing responsibility further allows us to maintain momentum with group activities while respecting the ebbs and flows of individual members’ time and creative resources. This nourishing context feeds both our leadership collective and our individual needs.

*Pollinating—exchanging with others to enrich and diversify* The Careoperative provides an inclusive and trusting space for open pollination of ideas, tools and experiences within and beyond the collective. Each of us brings diverse knowledge, life experiences and understanding from other contexts into the Careoperative. We have, for example, built on this diversity in a horizon-scanning exercise to identify research frontiers in relation to future food system transformations that feed into joint proposal writing. The experience within the Careoperative has in turn inspired a number of us to open discussions on how to embed and support values of leadership collectives and care in our working environments and transformative change processes elsewhere. These forms of “pollination”—through development and exchange of how to work collectively—play an important role in enabling sustainability transformations.

*Seeding change—encouraging collective processes and actions to emerge* The Careoperative is founded on active, critical and collaborative reflection that encourages new ideas and approaches to emerge. We interviewed each other about what transformational leadership means to us to develop a shared understanding of transformational leadership. With this as a starting point, we collectively created a document describing the Careoperative Fundamentals (see Supplementary Material 1) that details our core vision, mission and values. This reference document elaborates the processes we follow to integrate shared values, collective responsibility and self-reflection into the way we work together, and helps us continuously consider the challenges of inclusivity. We are also working on developing a code of collaboration, further detailing decision-making processes, conflict prevention and resolution, and authorship policies.

External facilitation with a professional facilitator trained in process work and other facilitation methods has been invaluable for supporting our collaborative work and deep reflection. Based on the belief that the means to reach the objectives are key elements in transformational work and for effective group work (Schwarz 2002), the facilitator’s role has been to accompany the group to formulate and reach its goals, while demonstrating care of people in the group, as well as the process. The facilitator guided our group whilst sharing insights to the innovative methodologies used, such as social technologies (open space, world cafe, pro-action cafe, etc.), future scenario planning, process work techniques, etc., thereby developing our own facilitation skills. Critical reflection is a further vital function that inspires us to do things differently, create alternatives to the dominant work culture of academia, and strive towards sustainable social change. Attention to processes and critical reflection has facilitated activities that lead to tangible outputs, including funding applications, workshops and writings.

## Leadership collectives to enable sustainability transformations

There is broad agreement that more effective leadership for sustainability transformations is needed, and that academia should play an important role in such transformations by training future transformational leaders, and by contributing to societal knowledge brokering processes. But structural barriers within academic funding and reward systems arising from a systemic focus on individual excellence and leadership for high-pace productivity within academia make such transformational academic leadership difficult. Breaking down barriers that reinforce incentives for individual leadership can only be done by reshaping and diversifying the academic spaces in which we operate. Thus, fostering a caring, inclusive, merit-oriented, truly inter- and transdisciplinary academic space requires re-orienting training programs, work ethics and reward systems.

Leadership collectives provide both a mechanism and outcome for achieving more effective leadership for sustainability transformations. As a mechanism, leadership collectives can support a move in academia from metrics to merits, from a focus on career to care, and enact a shift from disciplinarily-bounded to inter- and trans-disciplinary research. As an outcome, our living experiment of the Careoperative provides one example of what a leadership collective can look like. We strive for deep positive change in ourselves, our academic relationships and (academic) culture, which is critical for scaling collective leadership for change. We do this work with shared values rooted in critical reflection, inclusivity and care.

We encourage other institutions and initiatives to provide researchers and practitioners with the space and time to develop relationships and cooperation based on care. Most importantly, our group has provided a source of hope, energy and support to continue our collective discovery of leadership in sustainability transformations research. We call on the generations of leaders who have come before us, including current senior leaders of established academic institutions working in sustainability research, to cultivate spaces where leadership collectives can flourish and future leaders can work together to enact radically reimagined visions for sustainability transformations.

## Supplementary Information

Below is the link to the electronic supplementary material.

Supplementary file1 (DOCX 23 KB)

## Footnotes

1 Postdoc Academy for Transformational Leadership funded by the Robert Bosch Stiftung https://www.bosch-stiftung.de/en/project/postdoc-academy-transformational-leadership.
