# Supplementary material for: Creating leadership collectives for sustainability transformations
Source: Sustain Sci. 2021 Mar 4;16(2):703–8. doi: 10.1007/s11625-021-00909-y (PMC7929730; doi:10.1007/s11625-021-00909-y)
Supplement: Supplementary file 1 — Supplementary file1 (DOCX 23 KB) [file 11625_2021_909_MOESM1_ESM.docx]

**Electronic Supplementary Material**

**Article: Creating leadership collectives for sustainability transformations**

**Supplementary Material 1 - The Careoperative Fundamentals**

*Last updated: 21.12.2020*

**We are the** *Transforming (food) Systems Careoperative*

The Careoperative is a leadership collective which we define as a group of individuals from multiple organisations and sectors who lead transformational social change together through critical reflection, inclusivity and care.

As a **careoperative**, we focus on strong social relationships and solidarity - being responsible to one another and sharing in the costs and benefits of membership. It is a caring space.

Our **vision** is to create a long-term space of support for each other.

Our **focus** is transforming (food) systems for sustainability.

Our **mission** as a careoperative is to explore, embody and pollinate transformational sustainability and transdisciplinary research.

Our **shared values** in relation to (food) systems are sustainability, equity, quality, access.

Our **shared values** in relation to the careoperative are inclusiveness with multiple perspectives; solidarity, transparency.

Our **shared values** in relation to leadership are care, inclusivity and critical reflection.

We anticipate that **outputs** will emerge as the means to achieve our vision and mission. Outputs include but are not limited to position papers, opinion pieces, mentoring, training, etc. We aim to **achieve** more-than-outputs.

As more-than-transformational researchers, our **activities** include:

- Meeting together
- Sharing professional and personal experiences
- Connecting different perspectives and scales
- Plurality of leadership
- Being and finding allies
- Being and finding role models
- Being pollinators - sharing ideas to take and use in our own contexts.

We are **radical** in our pursuit of transforming [food] systems, in as much as radical means:

- Going to the root of problems
- Being care-active in the way we construct and deconstruct systems
- Being critical, reflexive and mindful, in how we work over time

We engage in periodic **reflection** on the type of transformation we seek.

**Membership** of this Careoperative is the first cohort of the Robert Bosch Foundation Postdoc Academy for Transformational Leadership. Those belonging to this group receive inward focused support. They are to be kept informed of all Careoperative activities and have the opportunity to join in. Participation is on an opt-in basis. No one is required to participate in anything, and not everyone will do everything.

The Careoperative members **collaborate** with others for outward looking activities.

The Careoperative has a group of **Caretakers** who help to coordinate its activities.

**Ways of Working**

*Caretakers*

These are four people chosen annually by the members through Sociocratic Elections^^[[1]](#footnote-1)^^. This system, together with all other collective processes are to be reviewed annually by the Careoperative.

The **Caretakers** are responsible for:

- Maintaining an overview of ongoing activities and ensuring it is accessible to all.
- Making sure regular meetings and communications happen
- Initiating an annual review of governance systems and activities and to enable group reflexivity
- Keeping track of agreed actions and making sure they are followed through.
- Handling complaints and conflicts procedures (as detailed in the Code of Collaboration).
- Overseeing the Careoperative’s care support functions: collective emotional management, support ebbs and flows of involvement and mediate in conflicts
- Overseeing liaison with external partners /funders

*Ebbs and Flows*

The Careoperative recognises that ebbs and flows in involvement are inevitable, and seeks to provide a caring context to accommodate this.

- It is fine not to be available for a short or long period. It is also fine to withdraw from activities that you said you would be involved in. In this case, please make sure someone involved in the activity knows.
- Prior to significant activities or meetings, we will use a ‘bridging’ process where members, who have not been involved recently, will be approached by another member to be updated and to help them reengage.

*Care Support*

- We want to acknowledge the importance of emotional support as part of this group and have in place ways to achieve this. We name this Care Support. This includes group dynamics, noting varying engagement / helping people drop in and out, using regular emotional support and contact to help avoid conflict.
- This is everyone’s responsibility with the Caretakers having additional attention to how it happens.
- Caretakers have the role of Ombudsperson - someone to approach if a member of the group is not happy with something / about a conflict.
- If someone is not happy with a Caretaker or would prefer not to approach them then go to anyone else in the group and ask them to raise it anonymously.
- All meetings will include a check-in to monitor personal and emotional aspects.
- The cycle of meetings includes Care Support focused meetings as space to discuss individual challenges or topics people want to explore. Anyone can propose a topic.
- If we notice someone has been out of contact for a while, check in with them on a one-to-one basis.
- A Mid-term Review will explore how things are working, including Caretakers roles.
- The Code of Collaboration details the values we share, and some ways we can enact them.

*Administration and project management*

- We are self managing and organising as this is part of being a transdisciplinary researcher and Careoperative.
- Slack is our main communication method.
- Email can be used for time sensitive/urgent communication relevant to everyone or to specific subgroup members.
- Each Slack channel should be kept updated with progress on an activity.
- We will seek to learn from best practice on how project management is done and develop ideas of what we mean by project management.
- We keep track of activity via a shared Google Sheet.
- In the medium to longer term we plan to develop a website or space where we communicate externally what we have done and share our internal documents.

*Meetings*

- The Careoperative has a weekly Zoom meeting scheduled to alternate between afternoon and morning to accommodate different time zones, and caring responsibilities.
- The focus of weekly meetings will rotate between:
  - Business - getting shit done
  - Care support - open space for dreaming/ visioning, trouble-shooting, personal support
  - Social - having fun and being in touch
- Meeting details, including agenda are recorded in the TO DO Google Sheet.
- Anyone can volunteer as Notetaker or Facilitator in order to rotate responsibilities.
- All meeting arrangements can be reviewed at the Annual and Mid-Term Reviews.

*Mid-Term Review*

- Every 6 months the Careoperative will review its working. This will explore what is working well and what is not working well.
- This is also an opportunity for Caretakers to share reflections on their roles and responsibilities.

*Annual Review*

- Every 12 months the Careoperative will review its working. This will explore what is working well and what is not working well.
- This is also when the selection of Caretakers takes place.

*Election of Caretakers*

The annual selection of the Caretaker team will take place using a Sociocratic Election Process, held with participation of all members of the Careoperative:

1. Prepare the Job Description (see above).
2. Open nominations for a set period:
3. Everyone writes three nominations ‘x nominates x’. You can nominate yourself. Do not discuss nominations until after the closing time.
4. Volunteers collate the nominations and share the results.
5. Facilitated Election Discussion
   a. Opportunity to explain your nomination – focus on why they are suitable
   b. Opportunity to withdraw nominations
   c. Discussion of nominations – focusing on past experience not what someone might do in future
   d. Candidates accept or decline nominations
6. Group consensus to endorse the nominated candidates.

1. <https://www.sociocracy.info/the-sociocratic-election-process/> [↑](#footnote-ref-1)
